# Flat telescope based on an all-dielectric metasurface doublet enabling polarization-controllable enhanced beam steering

**DOI:** 10.1515/nanoph-2021-0609

**Published:** 2021-12-08

**Authors:** Hongliang Li, Changyi Zhou, Woo-Bin Lee, Duk-Yong Choi, Sang-Shin Lee

**Affiliations:** Department of Electronic Engineering, Kwangwoon University, Seoul, 01897, South Korea; Laser Physics Centre, Research School of Physics, Australian National University, Canberra, ACT 2601, Australia

**Keywords:** beam steering, deflection magnification, flat telescope, metasurface doublet, polarization control

## Abstract

A flat telescope (FTS), which incorporates an all-dielectric metasurface doublet (MD) based on hydrogenated amorphous silicon nanoposts, is proposed and demonstrated to achieve flexibly magnified angular beam steering that is sensitive to both light polarization and deflection direction. Specifically, for transverse-electric-polarized incident beams, the MD exhibits deflection magnification factors of +5 and +2, while for transverse magnetic polarization, the beam is steered in reverse to yield magnification factors of −5 and −2 in the horizontal and vertical directions, respectively. The proposed MD comprises cascaded metalenses, which can invoke polarization-selective transmission phases. The MD which emulates a set of convex and concave lenses renders positively increased beam deflection, whereas the case corresponding to a pair of convex lenses facilitates negatively amplified beam deflection. The essential phase profiles required for embodying the MD are efficiently extracted from its geometric lens counterpart. Furthermore, the implemented FTS, operating in the vicinity of a 1550 nm wavelength, can successfully enable enhanced beam steering by facilitating polarization-sensitive bidirectional deflection amplifications. The proposed FTS can be applied in the development of a miniaturized light detection and ranging system, where the beam scanning range can be effectively expanded in two dimensions.

## 1 Introduction

Light beam steering, which has emerged as a core technology of the Fourth Industrial Revolution, has widespread applications in various fields, including light detection and ranging (LiDAR) [1], free-space optical communication [2], and virtual/augmented reality displays [3]. As a technology for enabling two-dimensional (2D) beam steering in a LiDAR system, a solid-state optical-phased array (OPA) based on liquid crystals and planar lightwave circuits has attracted significant attention as a viable alternative to its mechanical counterpart, offering the advantages of a fast and reliable response, compact footprint, planar thin structure, and affordable manufacturing [4], [5], [6], [7]. However, the conventional OPA has a major limitation in the field of view, which is a key component of beam steering [8], [9], [10].

Geometrical optics-based components, which were mainly prepared by matured mechanical machining, played a crucial role in constructing Risley prisms, spherical mirrors, and lenses [11], [12], [13]. The telescope, which typically consists of a pair of lenses, has been efficiently employed to achieve magnified angular beam steering [14]. Notably, a solid-state OPA combined with a telescope module has been demonstrated to achieve beam scanning, characterized by a substantially enlarged field of view [15]. However, the telescope module, which was embedded in a bulky jig, was only able to increase the vertical steering range by a fixed factor. A telescope module using anamorphic aspheric lenses was recently developed to allow for different magnification factors based on the beam steering direction, offering improvements in compactness, weight, and integration [16, 17].

Various optical metasurfaces, which manipulate the wavefronts of lightwaves in terms of the amplitude, phase, and polarization, have been extensively researched for a wide range of applications, including biomedical devices [18, 19], holographic devices [20, 21], and metalenses [22], [23], [24], [25], [26]. Unlike geometric optics-based components, metadevices capitalizing on subwavelength polarization-sensitive meta-atoms can potentially offer security encryption [27], polarization multiplexing [28, 29], or diversified functionalities [30], [31], [32]. A nanograting-based telescope was developed for amplifying the beam propagation angle [33]. However, only a single low magnification factor was realized as a result of the complex device design and delicate processing technology. Considering that the performance of a conventional OPA is mostly determined by the beam scanning range [9], variable angular magnifications in the scanning direction are preferable. From the perspective of developing a state-of-the-art LiDAR system, a polarization-controlled metasurface platform would likely offer advanced beam steering features through polarization adjustability and direction-selective angular beam magnifications.

Therefore, the objective of this paper is to develop an all-dielectric metasurface doublet (MD)-based flat telescope (FTS), incorporating an array of hydrogenated amorphous silicon (a-Si:H) nanoposts on either side of a silica spacer, for enhancing beam steering, in terms of multiple angular magnifications through the adjustment of the beam steering direction and light polarization. Specifically, the objective of the FTS is to enable bidirectional (positive and negative) deflections under transverse electric (TE) and transverse magnetic (TM) polarizations, respectively. Meanwhile, the designed FTS achieves different magnification factors in the horizontal and vertical directions. The developed MD, which can be manufactured via electron beam lithography (EBL), mimics a telescope module drawing upon a pair of lenses. The MD corresponding to a set of convex and concave lenses enables positively amplified beam deflection, whereas the case corresponding to a pair of convex lenses provides negatively amplified beam deflection. For the proposed FTS, the phase profiles for the metalenses are efficiently derived from their geometric lens counterparts. As a proof of concept, the FTS is developed and operated successfully to achieve 2D angular beam magnifications at a telecommunications wavelength of 1550 nm.

## 2 Results and discussion

**Figure 1:**
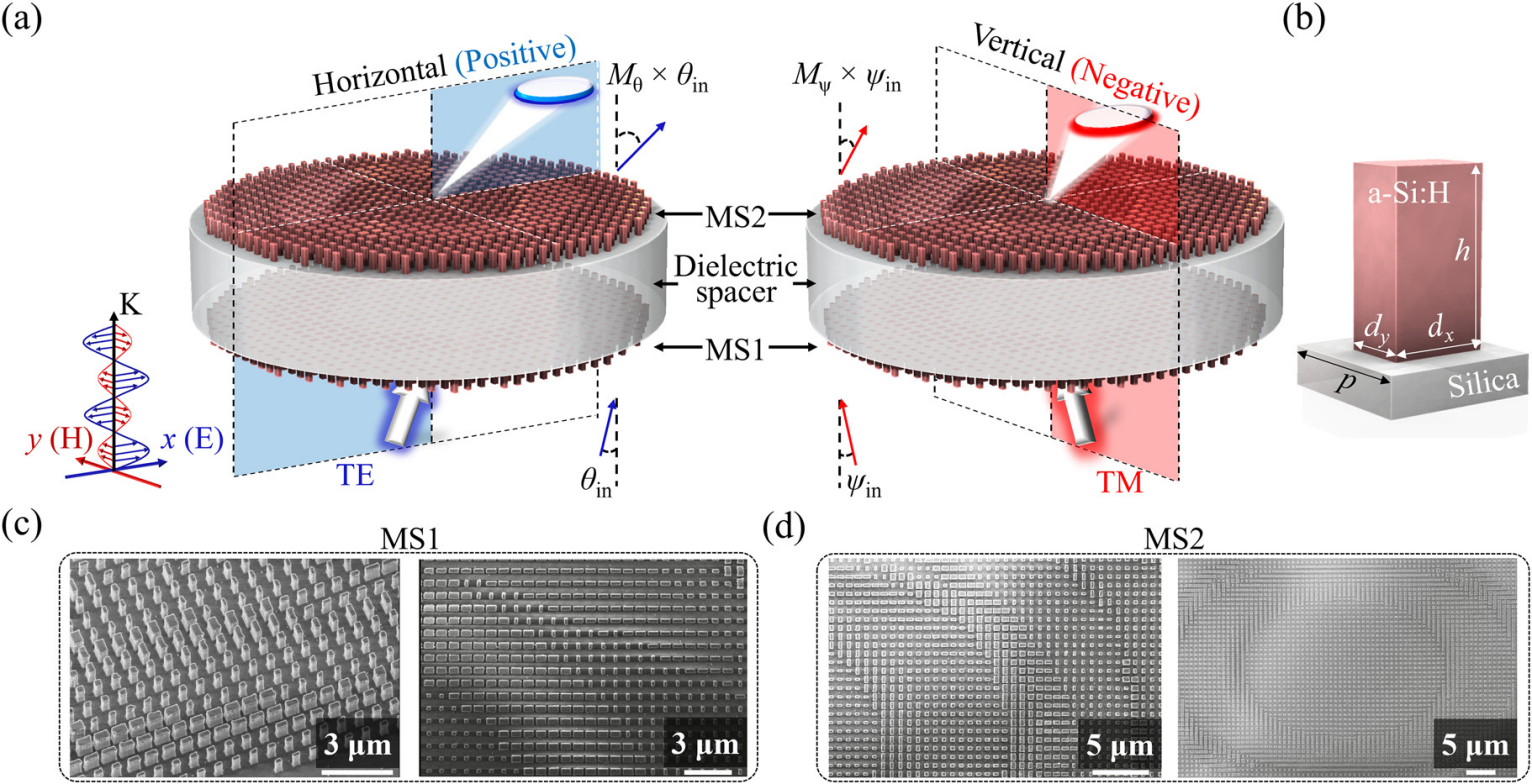
Configuration of the proposed FTS. (a) Illustrations of beam deflection mediated by the MD-based FTS under TE and TM polarization at a wavelength of 1550 nm. (b) Configuration of the designed a-Si:H nanopost formed on a silica substrate constituting the meta-atoms. (c) and (d) SEM images of the fabricated MS1 and MS2, respectively.

Figure 1(a) depicts the operation of the proposed MD-based FTS. It incorporates a pair of cascaded metasurfaces (MS1 and MS2) where an incident light beam deflects under TE and TM polarizations, with the electric field (E-field) oriented along the *x*- and *y*-axes, respectively. The FTS was constructed by integrating MS1 and MS2, acting as input and output lenses of the MD, respectively. The incident beam was deflected in accordance with different angular magnifications *M*, depending on the spatial direction. In particular, the beam was deflected to exhibit propagation angles *θ*_out_ and *ψ*_out_ along the horizontal (*x*-axis) and vertical (*y*-axis) directions, respectively, translating into *M*_θ_ and *M*_ψ_ angular magnifications. Furthermore, the developed FTS induced positively and negatively amplified beam deflections under the TE and TM polarizations, respectively. For the positive deflection, the deflected and incident beams were on opposite sides, with respect to the normal, whereas for the negative deflection, the two beams resided on the same side. Unlike conventional telescopes that use unidirectional steering, the developed FTS, which enables bidirectional beam steering without changing the incident beam direction, effectively doubles the achievable steering range. Figure 1(b) illustrates a schematic of a representative a-Si:H nanopost meta-atom constituting the two metasurfaces. This exhibits several key features, including low optical absorption [34, 35], a high refractive index in the vicinity of *λ* = 1550 nm (*n* = ∼3.45), and compatibility with complementary metal-oxide-semiconductor processes. A set of a-Si:H nanoposts with different cross-sectional dimensions of *d*_x_ and *d*_y_, which were designed to induce a polarization-dependent phase shift, were arranged in a square lattice to attain an entire 2π transmission phase and a high transmittance of over 80% (Section S1, Supporting Information). The period (*p*) of the lattice was 800 nm along both the *x*- and *y*-axes. The height (*h*) of the nanoposts was 880 nm. An a-Si:H nanopost-embedded MD, occupying a diameter of 500 μm, was created on either side of a silica substrate, serving as a dielectric spacer (*n* = 1.44), with a thickness of 902 μm. The fabricated MS1 and MS2 were inspected individually using a scanning electron microscope (SEM), as shown in Figure 1(c) and (d).

**Figure 2:**
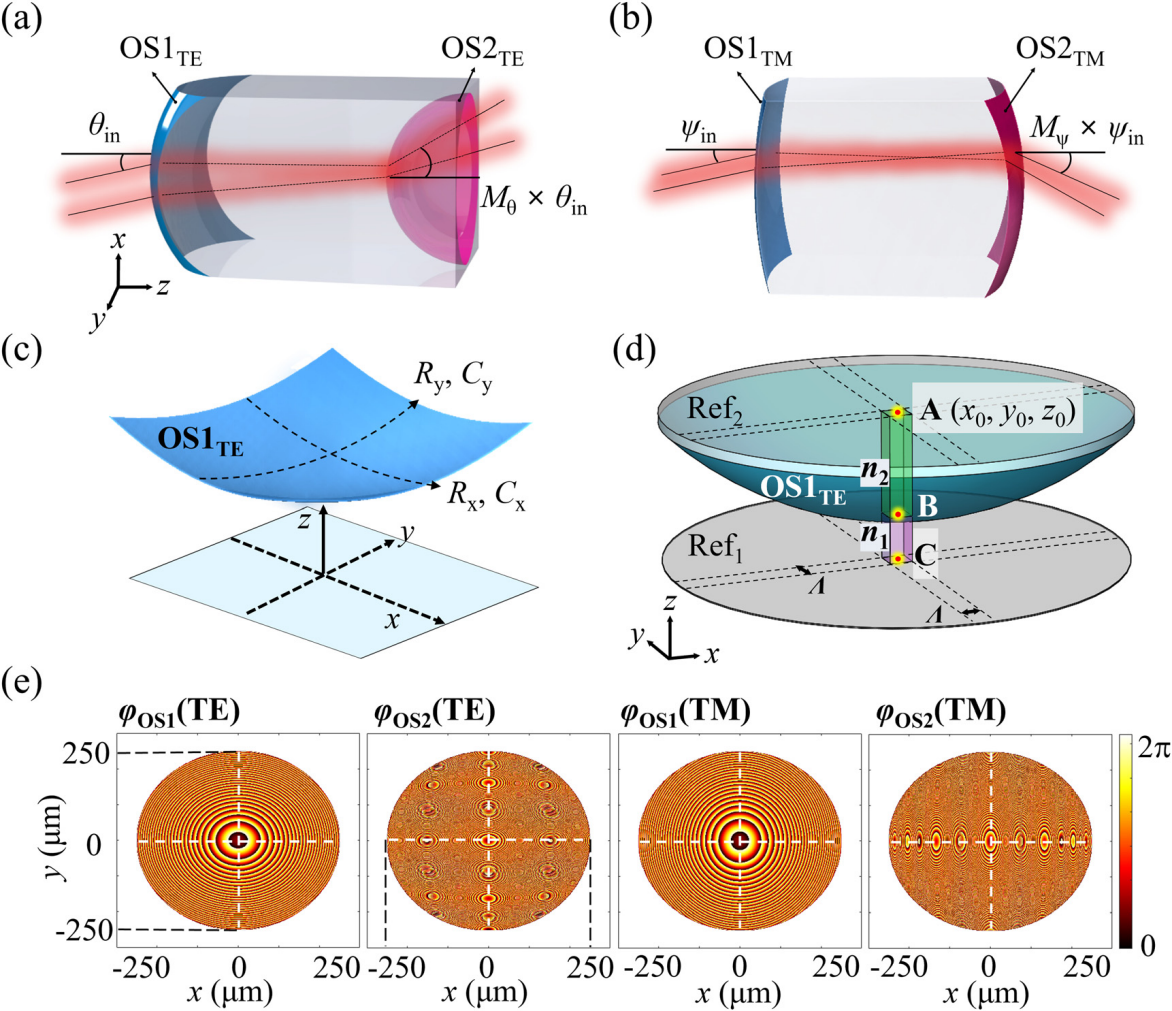
Design of an anamorphic lens doublet, involving phase profile calculation. Schematic configurations of lens doublets capitalizing on: (a) a convex contour OS1_TE_ and concave contour OS2_TE_, rendering increased beam deflection in the positive direction, (b) double convex contours OS1_TM_ and OS2_TM_, leading to increased beam deflection in the negative direction. (c) Perspective view of the anamorphic surface OS1_TE_ exhibiting different aspheric shapes in the *xz*- and *yz*-planes. (d) A local phase *φ*_lo_ at point A (*x*_0_, *y*_0_, *z*_0_), which is imparted to the incoming beam, is determined by the sum of the cumulated phase contributions from the paths connecting points C to B and points B to A, through different media. (e) Calculated relative phase profiles for OS1_TE_, OS2_TE_, OS1_TM_, and OS2_TM_.

The proposed FTS was designed in such a way that the required phase profiles for the MD were specifically extracted from the geometric lens doublets which emulate the configuration of conventional telescopes. Figure 2(a) and (b) show two conventional lens doublets, engaging four optical surfaces (OS1_TE_, OS2_TE_, OS1_TM_, and OS2_TM_), that were devised to boost the beam deflection in both the positive and negative directions. Moreover, each lens doublet allowed for different *M* factors along the horizontal (*x*-axis) and vertical (*y*-axis) directions, thereby resulting in distinct deflection amplifications. The constituting lenses were designed by conducting three-dimensional (3D) ray-optic simulations using LightTools (Synopsys) software. The lens doublet can be regarded as a combination of two separate lenses, as shown in Figure S2a and b (Section S2, Supporting Information). An identical focal plane is shared by the two lenses, and the effective focal length (*f*_1_) of the input lens is given as a factor of *M* for the output lens (*f*_2_) [36]. The doublet producing positive deflection was sequenced by convex and concave lenses, whereas the doublet producing negative deflection used two convex lenses. Specifically, OS1_TE_ and OS1_TM_ are responsible for diminishing the beam spot size, and the convergent beam starts diverging upon reaching OS2_TE_ and OS2_TM_, which are responsible for positive and negative beam deflections, respectively. The propagation angle of the deflected beam (*θ*_out_) is boosted by factors of *M* = +2, −2, +5, and −5 compared to the incident angle (*θ*_in_), where the sign of *M* represents the deflection direction. The strategy for designing the lens doublet has been outlined previously [33]. As depicted in Figure 2(c), to obtain unequal focal lengths in the horizontal and vertical directions, OS1_TE_ was designed based on an anamorphic aspheric surface. This surface exhibits bilateral symmetry along the *x*- and *y*-axes but does not always exhibit rotational symmetry [16]. Therefore, although the two radii (*R*_x_ and *R*_y_) are different, as in the cases of the conic constants (*C*_*x*_ and *C*_*y*_), the lens surface is still bilaterally symmetrical along the *x*- and *y*-axes. The anamorphic contour in the *xy*-plane is described in accordance with [17] as:

$$z\left(x,y\right)=\frac{\frac{1}{R_{x}}x^{2}+\frac{1}{R_{y}}y^{2}}{1+\sqrt{1-\left(1+C_{x}\right){\left(\frac{1}{R_{x}}\right)}^{2}x^{2}-\left(1+C_{y}\right){\left(\frac{1}{R_{y}}\right)}^{2}y^{2}}}$$

where *R*_x_, *R*_y_, *C*_x_, and *C*_y_ correspond to the designed OS1_TE_, OS2_TE_, OS1_TE_, and OS2_TE_, as listed in Table S1 of the Supporting Information. As plotted in Figure 2(d), the anamorphic surface OS1_TE_, as well as the two predetermined reference planes (Ref_1_ and Ref_2_), were applied to derive a local phase *φ*_lo_. To determine the phase required for creating the proposed metasurface, OS1_TE_ was spatially segmented into several small grid cells in accordance with a period *Λ*, while the center of each cell was taken as the origin. Here, the period *Λ* of the grid cells is equivalent to the period *p* of the meta-atoms made of a-Si:H nanoposts. The anamorphic surface OS1_TE_ was tailored to adequately manipulate the wavefront, from the perspective of the optical path lengths that each ray undergoes in the course of refracting from the lens-air interface. For instance, a ray of light intersecting Ref_2_ at point A (*x*_0_, *y*_0_, *z*_0_) accumulates a propagation phase for the path from point C (on Ref_1_) through B (intersected with OS1_TE_) to point A, contingent on the medium. Thus, the local phase *φ*_lo_ that a ray intersecting at point A accumulates can be expressed as:

$$\varphi_{lo}\left(x_{0},y_{0},z_{0}\right)=\int_{C}^{B}n_{1}k\text{d}z+\int_{B}^{A}n_{2}k\text{d}z$$

where *k* is the wavevector, and *n*_1_ and *n*_2_ are the refractive indices of the corresponding media [37]. The choice of the reference planes may affect the local phase *φ*_lo_ but not the relative phase *φ*_re_, which is determined by mod (*φ*_lo_, 2π). For the convex contour of OS1_TE_, *n*_1_ = *n*_air_ and *n*_2_ = *n*_lens_. For the concave contour of OS2_TE_, *n*_1_ = *n*_lens_ and *n*_2_ = *n*_air_. The phase profiles associated with the four optical contours, denoted as *φ*_OS1_(TE), *φ*_OS2_(TE), *φ*_OS1_(TM), and *φ*_OS2_(TM), are plotted in Figure 2(e). Hence, the angular deflection magnification, which is governed by the beam steering direction and light polarization, can be attained by tailoring the phase profiles encoded by the MD. This approach is highly customizable, given that the required phase profiles are extracted from a pre-designed geometric lens doublet, rather than the spherical/hyperbolic surface formula [38, 39].

**Figure 3:**
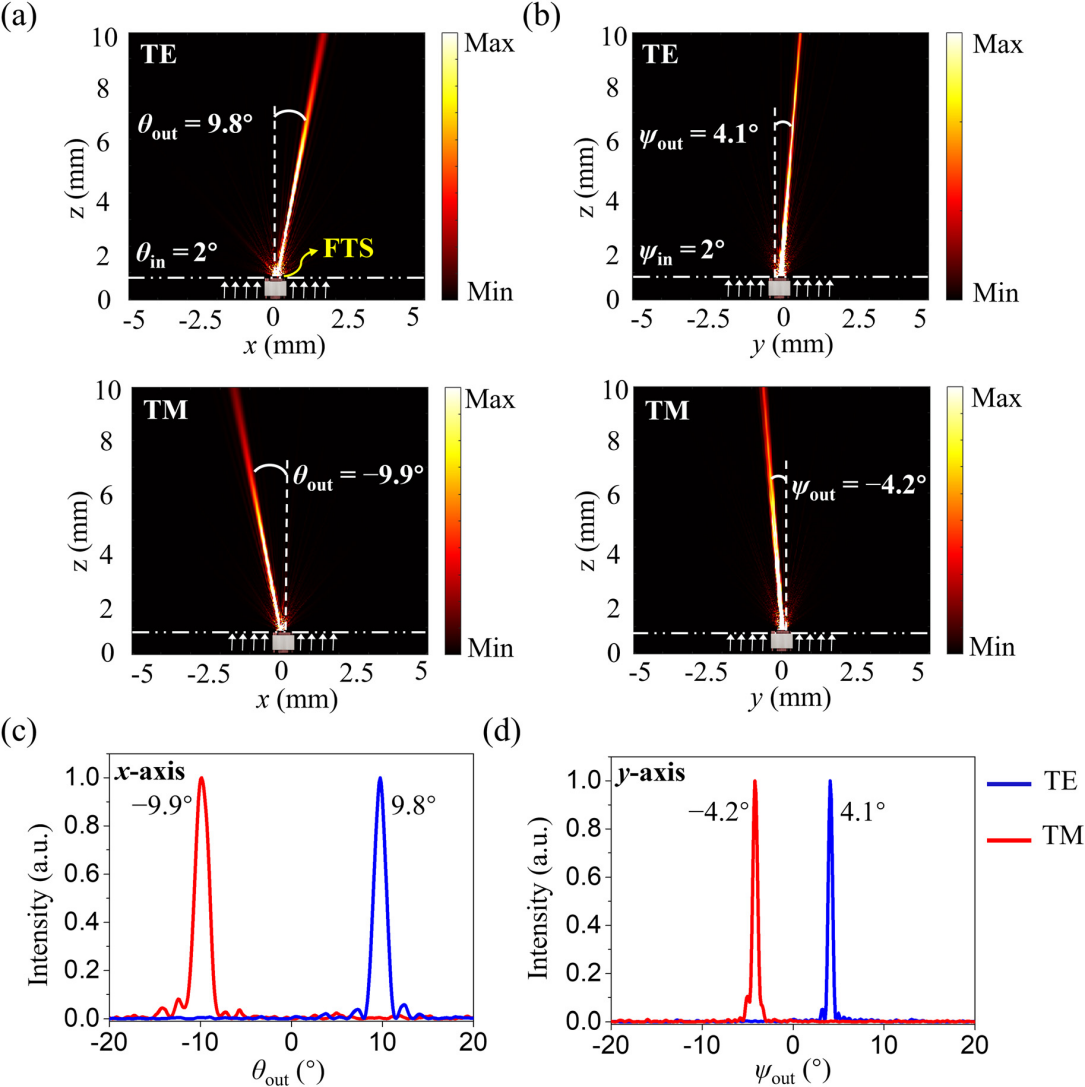
Simulation results for the developed FTS at a wavelength of 1550 nm: E-field distributions with *θ*_in_ = +2° and *ψ*_in_ = +2° in the (a) *xz*-plane and (b) *yz*-plane for TE and TM polarizations, respectively. The white arrows indicate the propagation direction of the incident beam. Far-field intensity distributions of the deflected beam along (c) *x*-axis and (d) *y*-axis.

To validate the operation of FTS, we conducted rigorous simulations using a commercial finite-difference time-domain software, FDTD Solutions (Ansys/Lumerical, Canada). The phase distributions corresponding to the central column and row, indicated by white dotted lines in Figure 2(e), were primarily considered (Section S3, Supporting Information). Figure 3(a) shows the calculated E-field distributions in the *xz*-plane for a deflected beam with *θ*_in_ = 2°. It is observed that the beam is deflected to render bidirectional beam steering, corresponding to angles of 9.8° and −9.9° for the TE and TM polarizations, respectively. Similarly, in the *yz*-plane, a beam with *ψ*_in_ = 2° is deflected to assume angles of 4.1° and −4.2°, as shown in Figure 3(b). In addition, the deflected beam propagates in the positive and negative directions for TE and TM polarizations, respectively, as expected. Figure 3(c) and (d) plot the calculated far-field angular distributions along the *x*- and *y*-axes. The deflection angles, *θ*_out_ and *ψ*_out_, are enlarged by factors of ∼±4.9 and 2.1 in the horizontal and vertical directions, respectively, conforming with the desired deflection amplifications of *M*_θ_ = ±5 and *M*_ψ_ = ±2. To explore its potential spectral range of operation, the performance of the proposed FTS has been calculated and compared for three different wavelengths including 1500, 1550, and 1600 nm (Section S4, Supporting Information).

**Figure 4:**
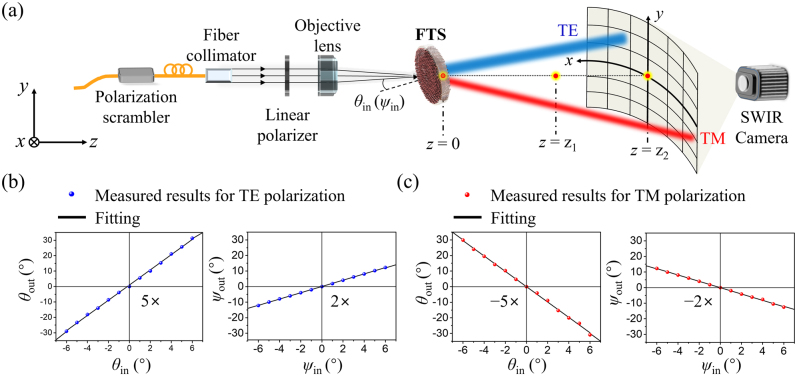
Transfer characteristics of the developed FTS: (a) Experimental setup for capturing output beams at various incident angles. Comparison between the experimental angular magnifications and fitted trajectories for (b) TE and (c) TM polarizations.

The developed planar FTS was evaluated using a custom-built test setup, as depicted in Figure 4(a). The light source was supplied by a distributed feedback laser (ALCATEL, A1905LMI), and a polarization scrambler (FIBERPRO, PS 3300) was employed to prohibit the influence of polarization fluctuations in the laser. A light beam was collimated by a fiber collimator, polarization-selected by a linear polarizer (Thorlabs, LPNIR050-MP2), and focused via an objective lens (Thorlabs, AC254-050-C-ML) onto the FTS, with a diameter of ∼250 μm. Under varying incident angles, a shortwave infrared (SWIR) camera (AVAL DATA, ABA-001IR) was mounted on a motorized linear stage and used to monitor the transmitted beam through the FTS. The angle of incidence was altered by manually tilting the FTS using a rotational stage. The angles of *θ*_meas_ and *ψ*_meas_ were estimated using the locations of the deflected beam and undeflected beam (UDB). The deflection angle is given by *θ*_out_ = *θ*_meas_ ± *θ*_in_ (or *ψ*_out_ = *ψ*_meas_ ± *ψ*_in_) according to the incident polarization (Section S5, Supporting Information). By moving the SWIR camera along the *z*-axis, the deflection angles under TE and TM polarizations were observed as shown in Figure 4(b) and (c), respectively. When the TE-polarized beam changed from −6° to +6° for both *θ*_in_ and *ψ*_in_, the deflection angle *θ*_out_ increased from −30° to +30° in the horizontal direction, and *ψ*_out_ varied from −12° to +12° in the vertical direction. Additionally, *M* is positive for TE polarization but negative for TM polarization. Figure 4 shows that the gradients of the linear fittings under TE polarization were +5 and +2 for *θ* and *ψ*, respectively, while those under TM polarization were −5 and −2 for *θ* and *ψ*, respectively. All gradients are in good agreement with the expected results of this study. Owing to the reliable deflection magnifications, the developed FTS produced beam steering ranges of ±30° and ±12° in the horizontal and vertical directions, respectively, when the incident angle was limited to ±6° in both directions. Although the FTS could handle a larger incident angle, the achieved steering range was practically constrained by the experimental setup in terms of the deflection angle and the shadow effect imposed by the camera. The maximum incident angle, which was limited by simulation results and the effective footprint of MS2, was approximately calculated as ±8° for the horizontal direction and ±20° for the vertical direction, thus leading to a steering range of 80° × 80°. Under incident angles beyond the maximum ranges, the FTS is prone to suffering from degradations in performance, manifested by unwanted noise and inaccurate angular magnification. The divergence angle of the deflected beam is enlarged in a similar manner to the deflection angle, thus rendering elliptical beam shaping (Section S6, Supporting Information). The deflection efficiency, defined as the ratio of the deflected optical power to the input optical power, was measured and discussed as described in Section 7, Supporting Information.

**Figure 5:**
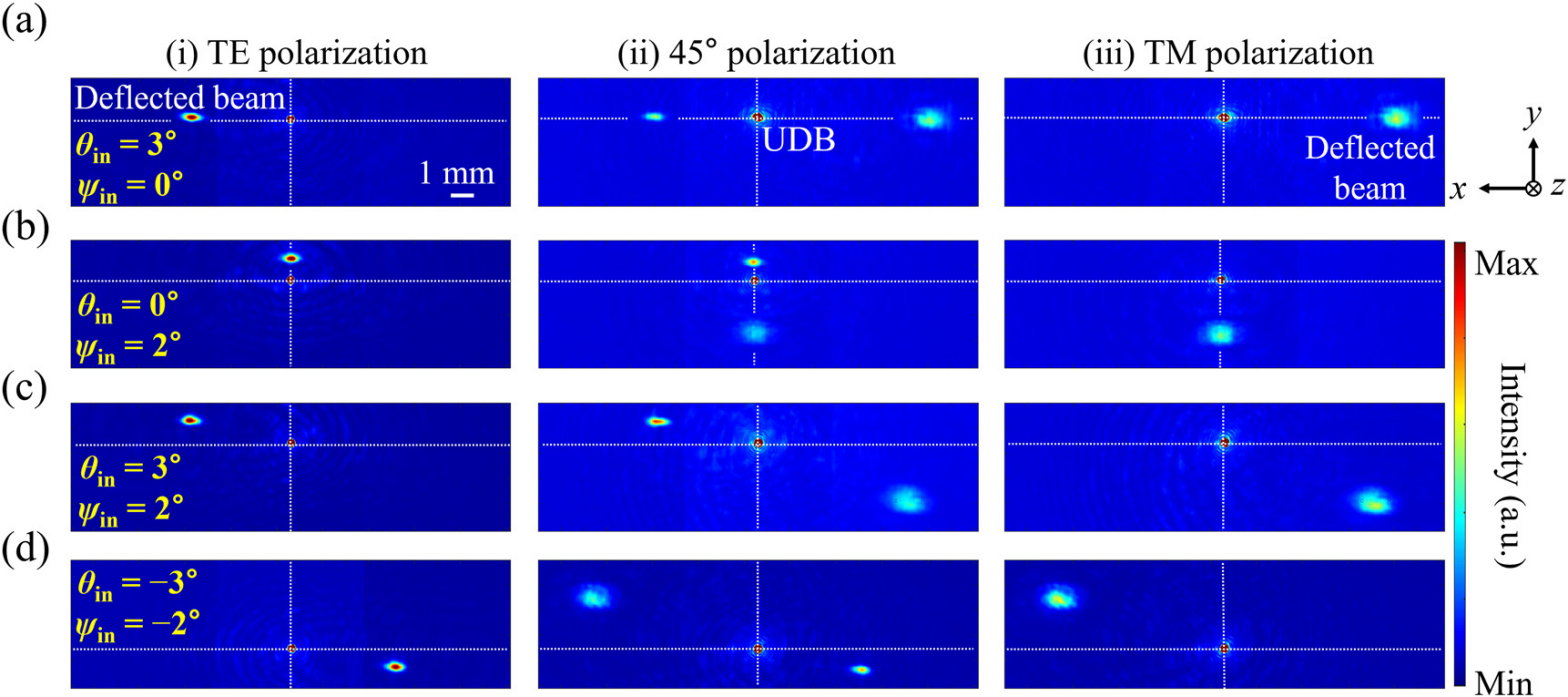
Characterization of polarization-tailored 2D beam steering: The incident angles *θ*_in_ and *ψ*_in_ are set at (a) 3° and 0°, (b) 0° and 2°, (c) 3° and 2°, and (d) −2° and −3° along the horizontal (*x*-axis) and vertical (*y*-axis) directions, respectively. The figures in the left (i), middle (ii), and right (iii) columns represent the TE, 45°, and TM polarizations, respectively. Each captured image has dimensions of 19.2 mm × 5.12 mm.

For beam scanning in LiDAR applications, metasurface-based angular magnification systems are widely accepted as a robust solution, in light of their ultra-thin form factor and flexible angular magnifications [40]. In this context, to investigate the feasibility of 2D beam steering, enabled by the proposed polarization-sensitive FTS, the output beam profiles were captured at *z*_1_ = 20.3 mm as measured from the MD, as shown in Figure 5. To record the output beams, the incident angles *θ*_in_ and *ψ*_in_ were set at ±3° and 0°, and 0° and ±2°, respectively, as shown in Figure 5(a)–(d). The TE-polarized beam, which was incident upon the FTS, was deflected along the horizontal and vertical positive directions according to the angular magnifications of *M*_θ_ and *M*_ψ_, respectively, while the TM-polarized beam was correspondingly deflected along the negative directions. The observed results for the case of 45° polarization are listed in column (ii). The beam was deflected along the positive and negative directions simultaneously to produce two distinct deflected beams, leading to an expected intermediate state between the TE and TM polarizations. The two steered beams corresponding to the orthogonal polarizations lie on the opposite side with reference to the normal rather than the UDB, as plotted in Figure S5 (Supporting Information). As shown in Figure 5(c) and (d), the incident beam, which is inclined with respect to the horizontal and vertical directions, is steered according to the corresponding *M*, providing a boosted 2D beam steering. Therefore, the beam scanning performance can likely be improved by flexibly expanding the scanning area through the adjustment of polarization.

## 3 Outlook and conclusion

With the aim of extending the range of angular magnifications, an MD, which is simultaneously playing the role of a pair of concave and convex lenses for both TE and TM polarizations, has been also designed to afford a larger scanning area. As shown in Figure S8 (Section S8, Supporting Information), customizable angular amplifications of +2, +4.7, +7.7, and +9.2 were achieved in simulations. The planar structure of the metalens-based FTS may help mitigate the adverse impact of total internal reflection relative to the curved surfaces of conventional lenses.

The proposed FTS may be legitimately flipped to manipulate light with a large angle of incidence. When light is reversely impinging upon it, the proposed FTS is categorically supposed to deliver an angular reduction factor of *M* [41, 42]. To verify this, the operation of the device has been investigated at a wavelength of 1550 nm by launching light toward MS2 (or OS2) instead of MS1 (or OS1), as shown in Figure S9 (Section S9, Supporting Information).

In summary, a miniaturized FTS operating at *λ* = ∼1550 nm, in which an all-dielectric MD based on a-Si:H nanoposts was built on a silica spacer, achieved polarization-controllable bidirectional angular magnifications. The incoming light beam was deflected bidirectionally through the adjustment of the incident polarization, assuming unequally magnified angles of propagation. The phase profiles in relation to the MD were efficiently acquired by imitating geometric lens doublet engaging anamorphic aspherical surfaces. The embodied polarization-sensitive FTS specifically enabled positive and negative magnifications of *M* = +2 and +5 and *M* = −2 and −5 in the horizontal and vertical directions, respectively. The prominent advantages, such as a miniaturized footprint, multiple angular magnifications, and polarization-controllability, allow the developed FTS to promote the development of advanced LiDAR and free-space communication transmitters/receivers.

## 4 Experimental sections

### 4.1 Device fabrication

The MD-based FTS was created on a 902 μm-thick silica substrate using a standard EBL process. The substrate, serving as a dielectric spacer, was cleaned using acetone, isopropyl alcohol, and deionized water in advance to promote its adhesion to the a-Si:H film. As shown in Figure S10 (Supporting Information), two 880 nm-thick films of a-Si:H were deposited using plasma-enhanced chemical vapor deposition (Plasmalab 100 from Oxford) on either side of the substrate. Next, a positive electron beam resist (ZEP520A from Zeon Chemicals) was spin-coated on one side of the substrate. Espacer (300Z from Showa Denko) was coated to prevent charging during electron-beam exposure. Subsequently, MS1 and alignment marks were created on the resist using EBL (Raith150) accompanied by a development in ZED-N50. Then, a 60 nm-thick aluminum film was deposited on the substrate via electron-beam evaporation (Temescal BJD-2000), and was patterned by lifting off the resist using a solvent (ZDMAC from Zeon Co.). The patterned aluminum was utilized as a hard mask during dry etching, thereby transferring the designed pattern to the underlying a-Si:H layer through fluorine-based inductively coupled plasma reactive ion etching (Oxford Plasmalab System 100). To eliminate the residual aluminum from the patterned nanoposts, wet etching was conducted. The sample was then flipped over to create MS2 by referring to both alignment marks on either side using a transmission optical microscope. MS2 was constructed by the same processes, including spin coating, EBL, aluminum deposition, lift-off, plasma etching, and aluminum removal. Note that misalignment between MS1 and MS2 could be readily overcome by adjusting the initial angle of the stage.

### 4.2 Measurement procedure

The test setup and procedure for assessing the completed FTS are shown in Figure 4(a). The FTS was precisely positioned by utilizing a visual fault locator (FIBERPIA, FP-VFL10N) along with a vision system. The angles (*θ*_meas_ and *ψ*_meas_) between the deflected beam and UDB were measured by manually scanning *θ*_in_ and *ψ*_in_ from 0° to ±6°. When the MD was rotated clockwise for the TE (TM) polarization, the beam was deflected toward the positive (negative) direction. The deflection angle was monitored by moving the SWIR camera along the *z*-axis.

### 4.3 Numerical simulations

(i) An optical software package, LightTools, was utilized to model the proposed FTS structure. The lens doublet, having an anamorphic aspheric surface, was modeled to achieve angular deflection magnifications including +2, +5, −2, and −5. (ii) As shown in Figure 3, the results of the E-field distributions were calculated using FDTD Solutions. For the design of the FTS device, a boundary condition of perfectly matched layer was adopted along the *x*- and *z*-axes, while a periodic boundary condition was adopted along the *y*-axis. In the 3D electromagnetic simulations, the MD has dimensions of 250 and 0.8 μm along the *x*- and *y*-axes, respectively, because of the limited computational resources. The source light with a wavelength of 1550 nm was assumed to impinge obliquely on the metasurface along the *z*-axis.

## Supplementary Material

Supplementary Material Details

Supplementary Material Details
