# Supplementary material for: Flat telescope based on an all-dielectric metasurface doublet enabling polarization-controllable enhanced beam steering
Source: Nanophotonics. 2021 Dec 8;11(2):405–13. doi: 10.1515/nanoph-2021-0609 (PMC11501665; doi:10.1515/nanoph-2021-0609)
Supplement: Supplementary file 1 — Supplementary Material Details [file j_nanoph-2021-0609_suppl.docx]

**Supporting Information**

Flat telescope based on an all-dielectric metasurface doublet enabling polarization-controllable enhanced beam steering

Hongliang Li^1^, Changyi Zhou^1^, Woo-Bin Lee^1^, Duk-Yong Choi^2^, and Sang-Shin Lee^1,^*

^1^Department of Electronic Engineering, Kwangwoon University, Seoul, 01897, South Korea

^2^Laser Physics Centre, Research School of Physics, Australian National University, Canberra, ACT 2601, Australia

*E-mail: slee@kw.ac.kr

1. **Optical transfer characteristics of the nanopost meta-atoms**

The transmission amplitude and phase of the nanopost meta-atoms for the TE and TM polarizations were calculated using a simulation tool, FDTD Solutions, as shown in Figures S1a and S1b, respectively. From the candidates of a-Si:H nanoposts formed on a silica spacer, with cross-sectional dimensions of *d*_x_ and *d*_y_ ranging from 200 to 650 nm, a set of 8×8 meta-atoms for MS1 and MS2, marked as white star symbols, was selected. This set of meta-atoms imparted an entire 2π phase shift for both TE and TM polarizations, yielding a transmittance of over 80% in response to a normally incident beam.


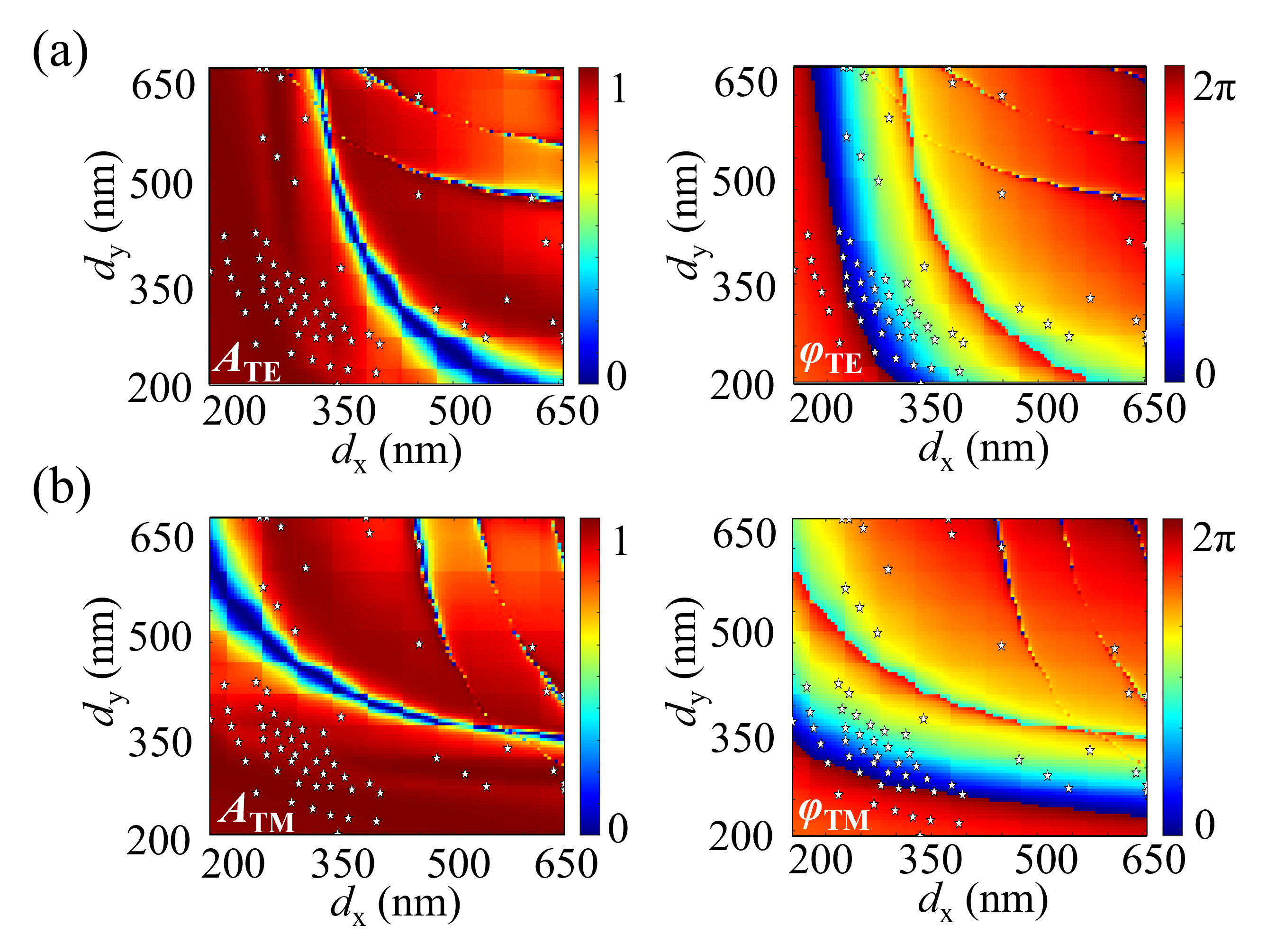


**Figure S1:** Optical characteristics of the nanopost meta-atoms under normal incidence: Simulated transmission amplitude *A* and phase shift *φ* of nanopost meta-atoms in terms of cross-sectional dimensions of *d*_x_ and *d*_y_ under illuminations of (a) TE and (b) TM-polarized beams, with a fixed height of *h* = 880 nm. White star symbols represent the selected meta-atoms.

1. **Operation of the modeled lens doublet**

Conventional telescope modules are typically known to adopt a focal system mimicking the Galilean or Keplerian telescope, where the focal points of two constituting lenses coincide. Figure S2a illustrates a lens doublet comprising a convex lens accompanied by a concave lens, leading to positive beam deflection, while Figure S2b pertains to the case incorporating two convex lenses, facilitating negative beam deflection. For each case, the two elemental lenses are deemed to share the same focal plane, and the effective focal length (*f*_1_) of the first lens is *M* times longer than that of the second lens (*f*_2_). Here, the focal length *f*_1_ of the input lens is positive. Meanwhile, the *f*_2_ of the output lens assumes positive and negative signs for the convex and concave lens, respectively, indicating that the focal plane resides inside or outside of the lens doublet. The magnification for the lens doublet is expressed as [1]:

$$M= \frac{\text{α}\text{2}\text{ }}{\text{α}\text{1}}\approx\frac{\tan^{-1}\text{α}\text{2}\text{ }}{\tan^{-1}\text{α}\text{1}}= \frac{\text{f}\text{1}\text{ }}{\text{f}\text{2}}.$$

The lens doublets delineated in Figures S2a and S2b resemble the Galilean and Keplerian telescopes, respectively. Parameters associated with the designed lens surfaces are listed in Table S1.


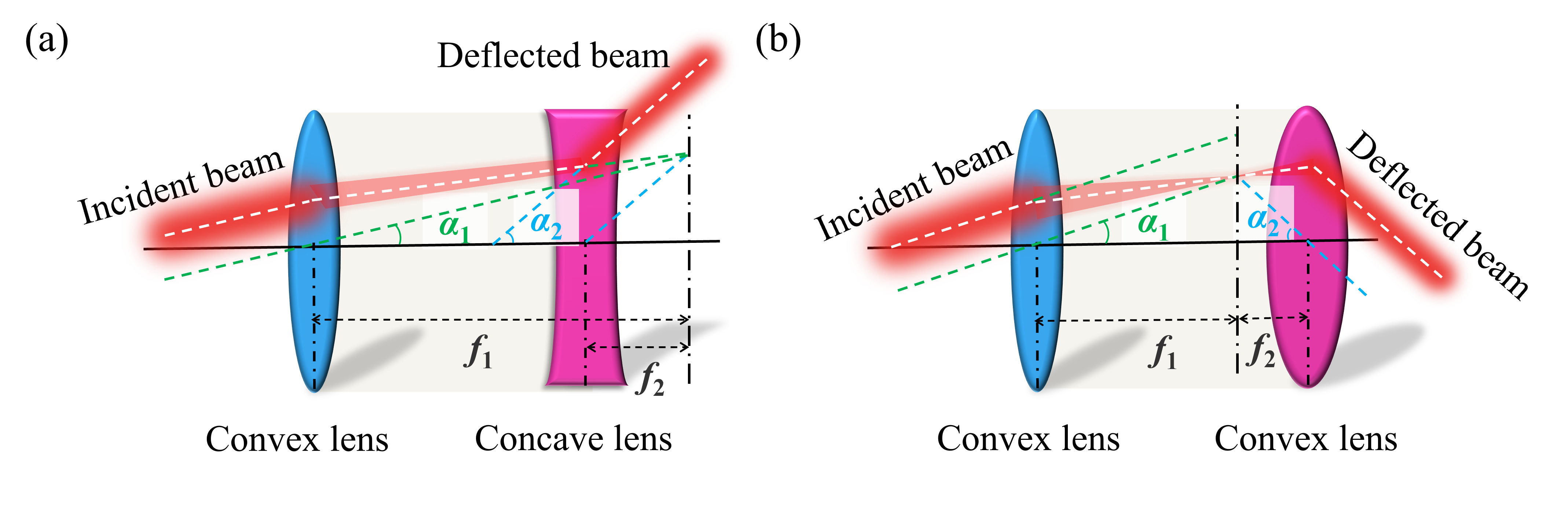


**Figure S2:** Operation mechanism of the lens doublets, incorporating (a) a convex lens followed by a concave lens and (b) a pair of convex lenses, elucidated in terms of the ray-optic behavior.

Table S1: Parameters for the contours of the four designed lens surfaces.

| OS | *Z* (*R*, *C*) | *R*_x_ [mm], *C*_x_ | *R*_y_ [mm], *C*_y_ |
| --- | --- | --- | --- |
| OS1_TE_ | $z(x,y)=\frac{\frac{1}{R_{x}}x^{2}+\frac{1}{R_{y}}y^{2}}{1+\sqrt{1-\left( 1+C_{x} \right){\frac{1}{R_{x}}}^{2}x^{2}-(1+C_{y}){\frac{1}{R_{y}}}^{2}y^{2}}}$ | 2.8998, −3.5 | 4.49, −5 |
| OS2_TE_ |  | 0.602, −0.25 | 2.195, 0 |
| OS1_TM_  OS2_TM_ |  | 1.9525, 0  1.57, 0.4 | 1.57, 0.4  0.778, 0 |

1. **Derivation of phase profiles for the developed MD**

For the MD comprising the proposed FTS, both theoretical and implemented phase profiles along the *x*- and *y*-axes for TE and TM polarizations, indicated by the white dotted line in Figure 2e, are illustrated in Figures S3a and S3b, respectively. Strong correlations between the desired and realized profiles imply that the MD-based FTS can manage polarization-controllable boosted beam steering.


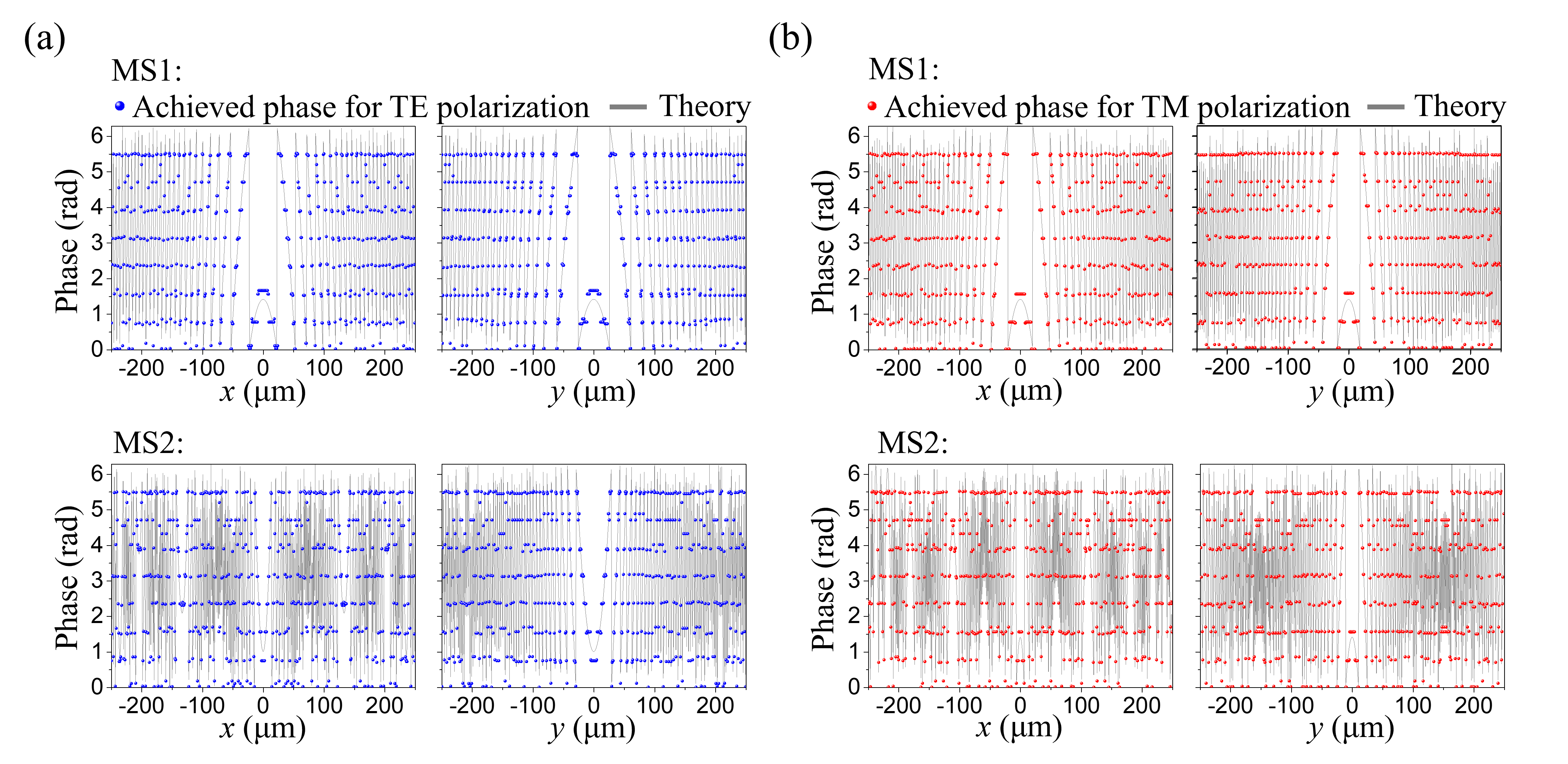


**Figure S3:** Theoretical and implemented phase profiles of the designed MS1 and MS2 for (a) TE and (b) TM polarizations.

1. **Beam deflection facilitated by the proposed FTS at near-infrared regimes**

The performance of the FTS has been specifically validated for different wavelengths in the near-infrared regime including 1500, 1550, and 1600 nm. The calculated far-field intensity profiles for the TE and TM polarization in response to an incident angle of 2° are shown in Figures S4a and S4b, respectively. It was confirmed that the deflection angle could be almost invariantly magnified as intended regardless of wavelength variations ranging up to 100 nm.


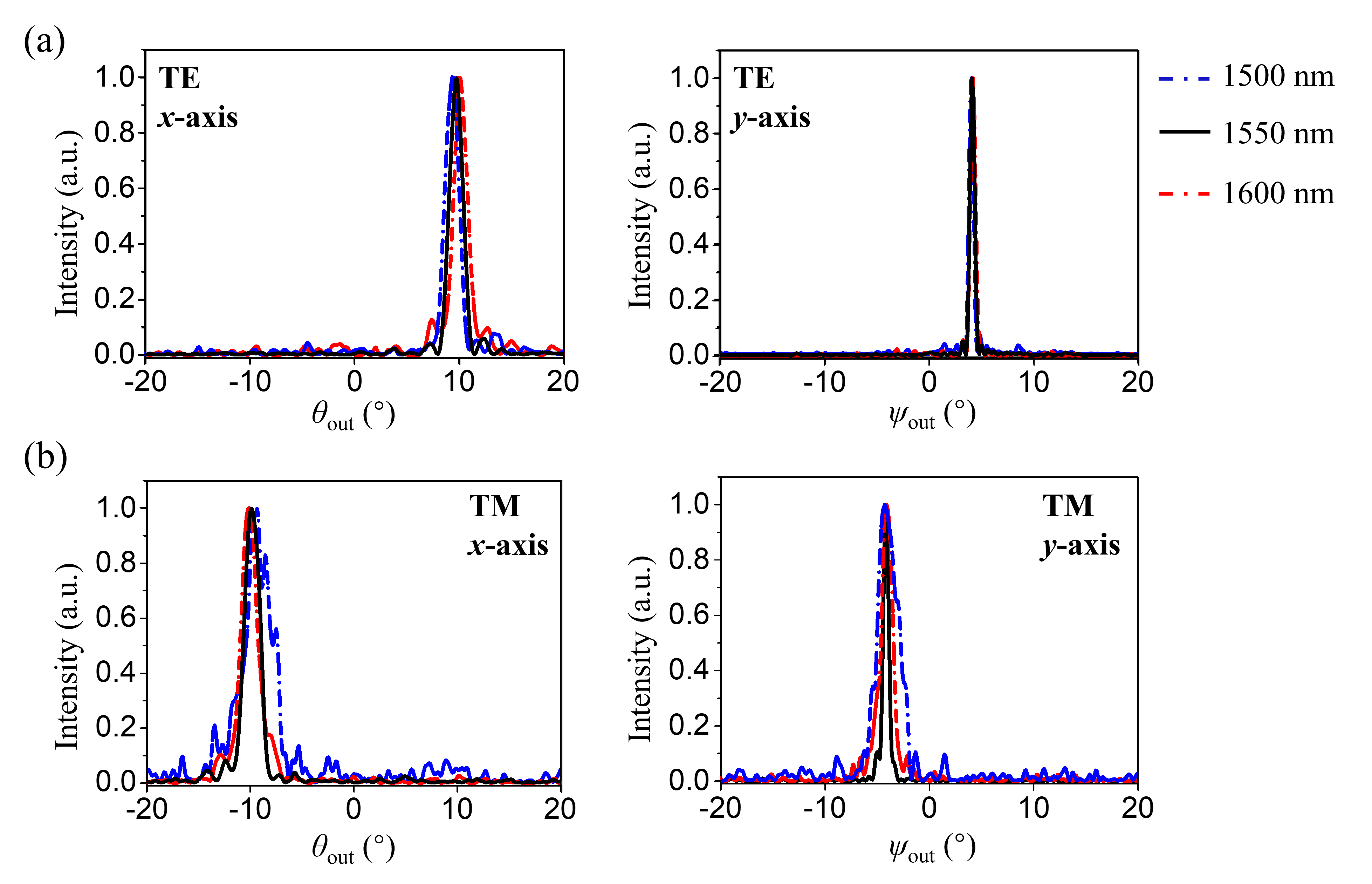


**Figure S4:** Calculated far-field intensity profiles for the proposed FTS with *θ*_in_ (or *ψ*_in_) = 2° at three wavelengths for (a) TE and (b) TM polarization.

1. **Description of *θ*_out_ (*ψ*_out_) and *θ*_meas_ (*ψ*_meas_) for positive and negative deflections**


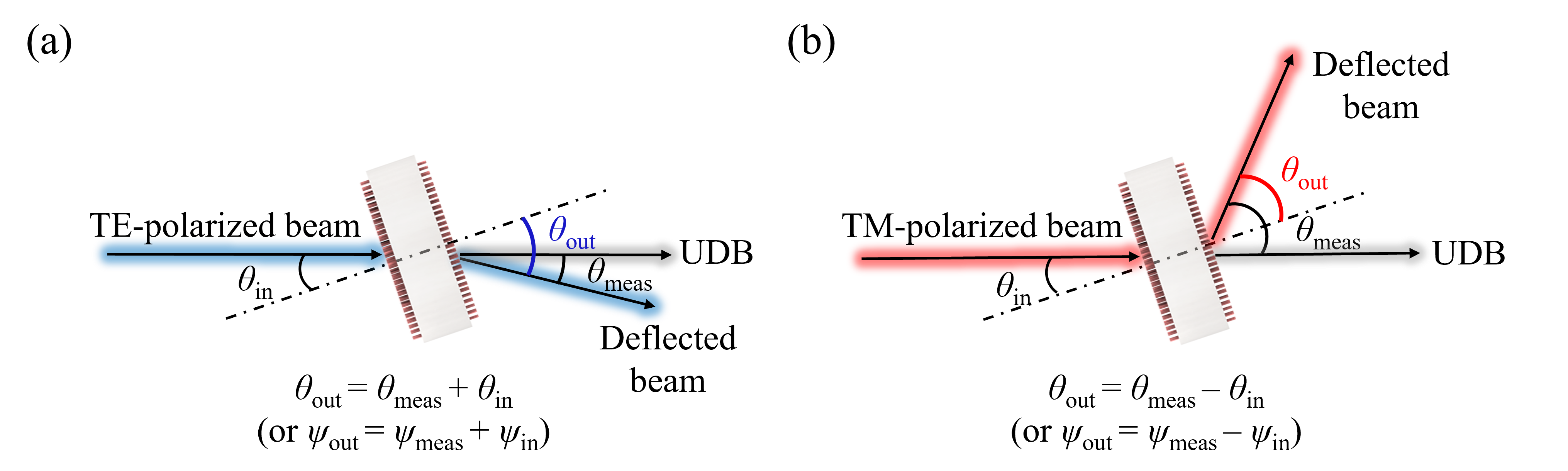


**Figure S5:** Schematics delineating the relationship between *θ*_in_, *θ*_out_, and *θ*_meas_ (*ψ*_in_, *ψ*_out_, and *ψ*_meas_) for the cases of a) positive and (b) negative deflections.

1. **Divergence characteristics of deflected beams**

Figures S6a and S6b present beam profiles recorded at two different positions, *z*_1_ = 20.3 mm and *z*_2_ = 30.3 mm, for the TE and TM polarizations, where the incident angles of *θ*_in_ and *ψ*_in_ are 3° and 2°, respectively. The divergence angle of the outgoing beam is expected to increase approximately at the same ratio as the deflection angle in the horizontal (*x*-axis) and vertical (*y*-axis) directions, rendering elliptical beam shaping. The magnification factor *M*, defined as *θ*_out_/*θ*_in_ and *ψ*_out_/*ψ*_in_, was approximately determined to be ±2 and ±5 from the gradient of the relationship, as shown in Figure 4. Here, the divergence angle, which is concurrently affected by the two lens surfaces, is inevitably enlarged. The intensity distributions along the dashed central lines, which pass through the center of the beams, are depicted in Figures S6c and S6d to calculate the divergence of the TE- and TM-polarized deflected beams, respectively. For positive deflection, the divergence angle of the steered beam is 2.52° and 1.15° along the horizontal and vertical directions, respectively, under an incident beam divergence of 0.52°. For the developed FTS, the beam divergence accordingly increases by factors of +5 and +2 for the TE polarization. Meanwhile, the deflected beam in the negative direction exhibits divergences of 5.6° and 2.51°, corresponding to 11- and 5-fold increments along the horizontal and vertical directions, respectively.

The discrepancy in magnification between the beam divergence and beam deflection for the TM polarization can be attributed partly to optical aberration and fixed substrate thickness. The contour OS2_TE_ of the first elemental lens, depicted in Figure 2a, can offset the partial aberrations caused by OS1_TE_. However, the combination of the two convex contours of OS1_TM_ and OS2_TM_, shown in Figure 2b, may unavoidably aggravate the aberrations. The performance of the embodied FTS may be alleviated by circumventing the aberrations by relying on a topology optimization framework [2]. Moreover, the gap between OS1_TM_ and OS2_TM_ progressively shrinks from the center towards the sides, while the substrate of the MD keeps a constant thickness of 902 μm; thus, MS1 and MS2 cannot accurately replicate the spatial distributions of OS1_TM_ and OS2_TM_ [3]. Owing to these shortcomings in the case of negative deflection amplification, the Galilean-type MD, which facilitates the positively increased beam deflection and better regulates the beam divergence, could be used in diverse applications.


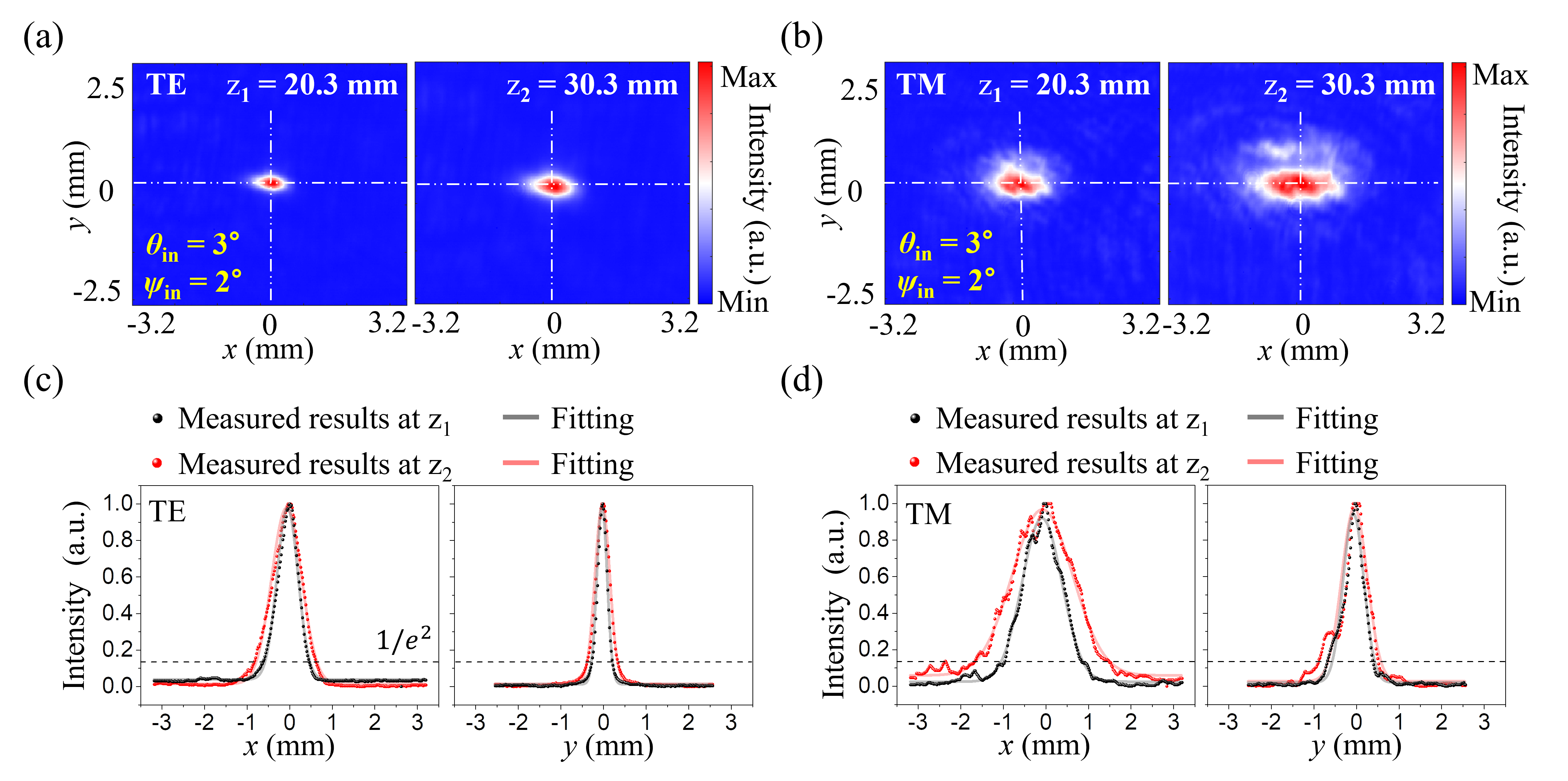


**Figure S6:** Beam profiles of the deflected beam captured at different positions: Intensity distribution in the *xy*-plane for (a) TE and (b) TM polarizations. Measured and fitted intensity distributions along the horizontal (*x*-axis) and vertical (*y*-axis) directions corresponding to (c) TE and (d) TM cases.

1. **Deflection efficiency of the proposed FTS**

The deflection efficiency of the proposed FTS, which depends on the light polarization and incident angle, has been monitored through a receiving photodiode (Thorlabs, S132C). Here, we were representatively concerned about a particular case of an incident angle of *ψ*_in_ = 2° for the TM polarization. The simulated deflection efficiency was 52.9%, which was higher than the demonstrated result of 13.2%. The discrepancy in efficiency between simulation and measurement might be mainly attributed to alignment and fabrication errors pertaining to the developed metasurfaces. Specifically, positional and angular misalignments between the constituting metasurfaces including MS1 and MS2 could possibly deteriorate the deflection efficiency [4−6]. Besides, errors in structural dimensions of the fabricated nanopost elements and the verticality of their sidewalls could adversely affect the transfer characteristics of the metasurfaces in terms of phase [7−9]. Moreover, the surface roughness of nanoposts was deemed to aggravate the scattering relating to the fabricated FTS, thereby incurring background noise [10]. It is anticipated that the proposed metasurface device can be substantially enhanced in terms of efficiency by substantially improving the fabrication processes and by possibly adopting an emerging scheme of deep learning [11,12].

1. **FTS enabling further enhanced beam deflection**


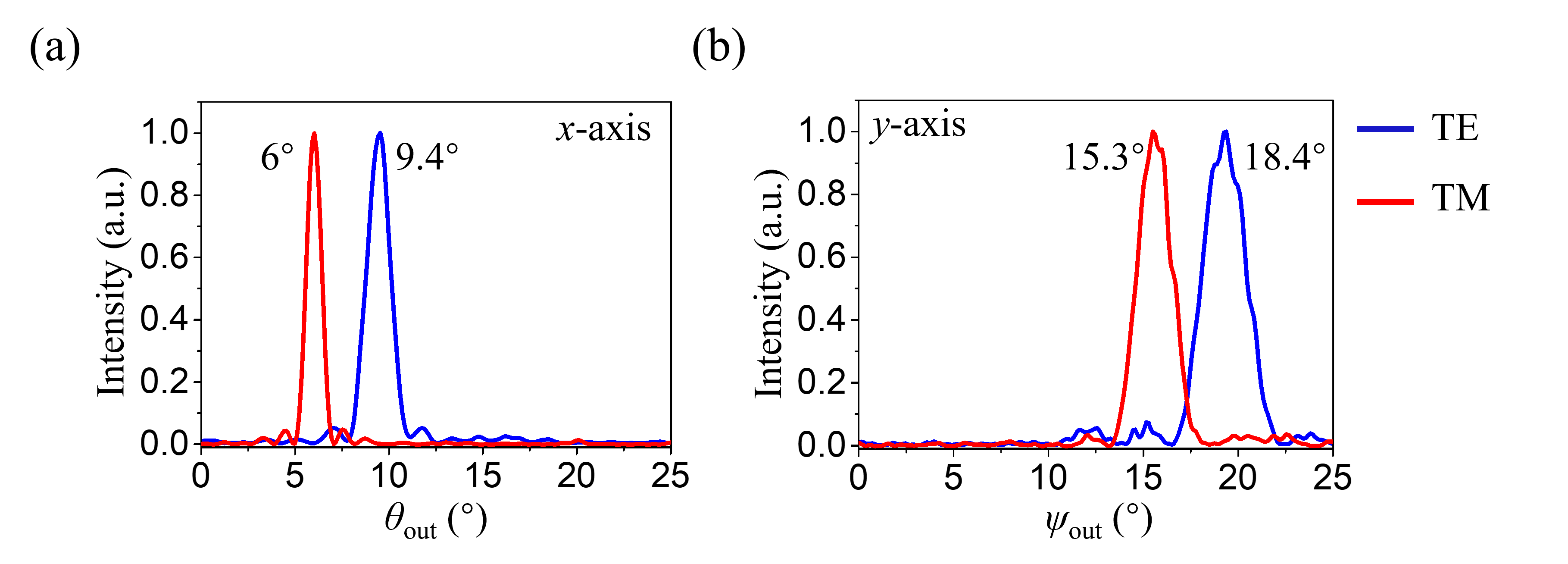


Figure **S8:** Calculated far-field beam profiles along (a) the horizontal and (b) vertical directions for the TE and TM polarizations enabled by the newly designed FTS, respectively. Here, *θ*_in_ = +2° and *ψ*_in_ = +2°.

1. **Response of the proposed FTS to reversely incident light**

Figure S9a shows the calculated E-field distributions in the *xz*-plane corresponding to the deflected beam in response to an incident beam with *θ*_in_ = −10° at a wavelength of 1550 nm. The propagation angle of the deflected beam is observed to diminish to −2° and 2° for the TE and TM polarizations, respectively. Figure S9b similarly shows that the deflection angle for *ψ*_in_ = −10° decreases to −4.9° and 4.9° in the *yz*-plane. Figures S9c and S9d plot the corresponding far-field angular distributions along the *x*- and *y*-axes. In case of incident light which is launched in the reverse direction, it is implied that the deflection angle can be approximately reduced by factors of ±5 and ±2 in the horizontal and vertical directions as expected, respectively.


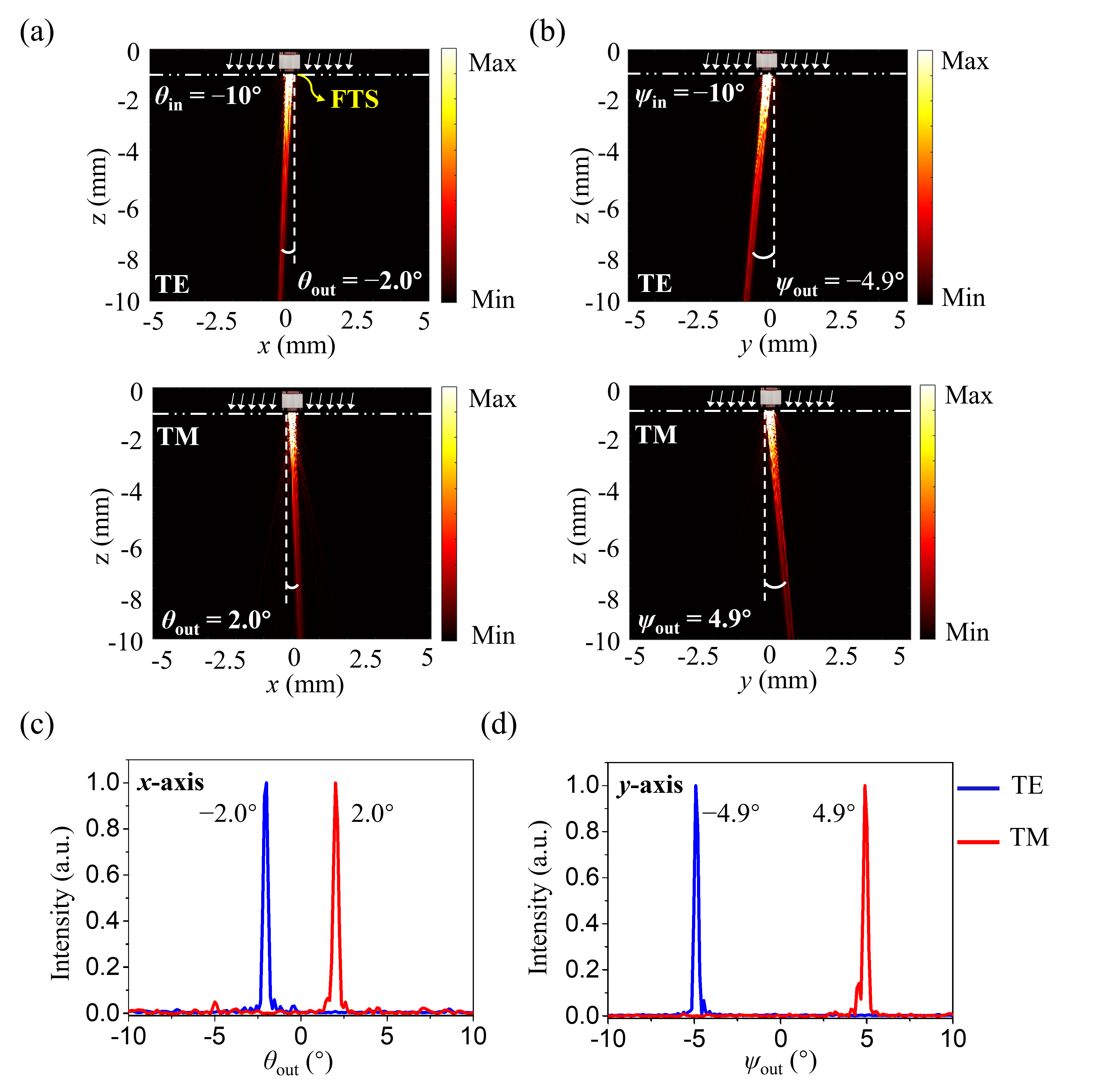


Figure **S9:** Calculated results for the developed FTS when the beam is launched from MS2 at a wavelength of 1550 nm. E-field distributions for the deflected beam with *θ*_in_ = −10° and *ψ*_in_ = −10° in (a) *xz*-plane and (b) *yz*-plane are observed for the TE and TM polarizations, respectively. The arrows in white indicate propagation direction of the incident beam. The corresponding far-field intensity distributions along the (c) *x*- and (d) *y*-axes are plotted.

1. **Nanofabrication**


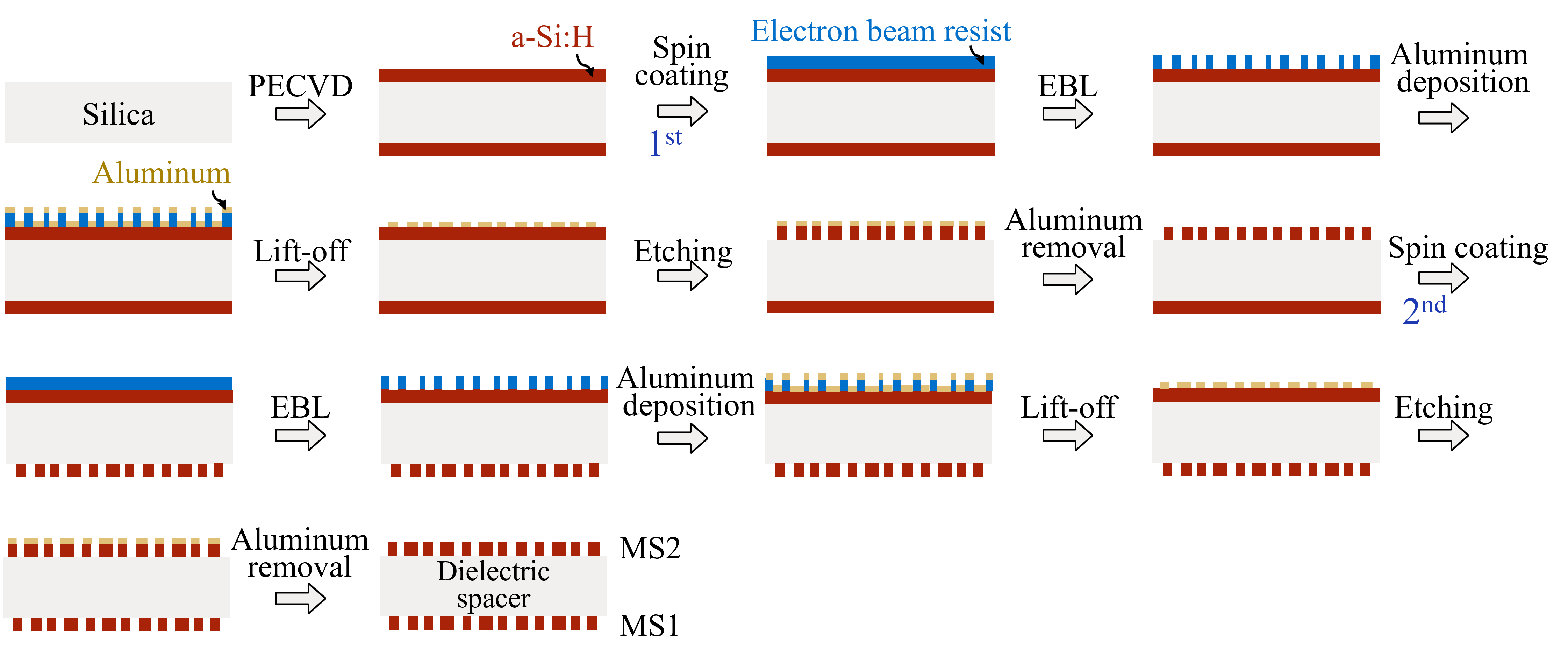


**Figure S10:** Fabrication procedure of the developed MD-based FTS.

References

1. G. Liu, Q. Lu, and W. Guo, “Ultrafast speed, large angle, and high resolution optical beam steering using widely tunable lasers,” *OSA Contin.*, vol. 2, pp. 1746–1753, 2019.
2. Z. Lin, B. Groever, F. Capasso, A.W. Rodriguez, and M. Lončar, “Topology-optimized multilayered metaoptics,” *Phys. Rev. Appl.*, vol. 9, p. 044030, 2018.
3. C. Zhou, W.-B. Lee, C.-S. Park, S. Gao, D.-Y. Choi, and S.-S. Lee, “Multifunctional beam manipulation at telecommunication wavelengths enabled by an all‐dielectric metasurface doublet,” *Adv. Opt. Mater.*, vol. 8, p. 2000645, 2020.
4. B. Groever, W. T. Chen, and F. Capasso, “Meta-lens doublet in the visible region,” *Nano Lett*., vol. 17, pp. 4902–4907, 2017.
5. Y. Zhou, I. I. Kravchenko, H. Wang, J. R. Nolen, G. Gu, and J. Valentine, “Multilayer noninteracting dielectric metasurfaces for multiwavelength metaoptics,” *Nano Lett*., vol. 18, pp. 7529–7537, 2018.
6. A. S. Backer, “Computational inverse design for cascaded systems of metasurface optics,” *Opt. Express*, vol. 27, pp. 30308–30331, 2019.
7. F. Silvestri, G. Gerini, S. M. B. Bäumer, and E. J. V. Zwet, “Robust design procedure for dielectric resonator metasurface lens array,” *Opt. Express*, vol. 24, pp. 29153–29169, 2016.
8. Y. Sun, L. Zhang, H. Xia, S. Cao, L. Wang, S. Yang, Y, Wu, and R. Tai, “Integrated silicon metasurface polarization beam splitter on a standard SOI substrate,” *Optik*, vol. 227, p. 166096, 2021.
9. B. Wang, F. Dong, H. Feng, D. Yang, Z. Song, L. Xu, W. Chu, Q. Gong, and Y. Li, “Rochon-prism-like planar circularly polarized beam splitters based on dielectric metasurfaces,” *ACS Photonics*, vol. 5, pp. 1660–1664, 2017.
10. A. Arbabi, E. Arbabi, S. M. Kamali, Y. Horie, S. Han, and A. Faraon, “Miniature optical planar camera based on a wide-angle metasurface doublet corrected for monochromatic aberrations,” *Nat. Commun*., vol. 7, pp. 1–9, 2016.
11. E. Arbabi, A. Arbabi, S. M. Kamali, Y. Horie, M. Faraji-Dana, and A. Faraon, “MEMS-tunable dielectric metasurface lens,” *Nat. Commun*., vol. 9, pp. 1–9, 2018.
12. R. P. Jenkins, S. D. Campbell, and D. H. Werner, “Establishing exhaustive metasurface robustness against fabrication uncertainties through deep learning,” *Nanophotonics*, 2021, Article ASAP, DOI: 10.1515/nanoph-2021-0428.
